# The Effects of Tai Chi Chuan on Improving Mind-Body Health for Knee Osteoarthritis Patients: A Systematic Review and Meta-Analysis

**DOI:** 10.1155/2016/1813979

**Published:** 2016-08-21

**Authors:** Wen-Dien Chang, Shuya Chen, Chia-Lun Lee, Hung-Yu Lin, Ping-Tung Lai

**Affiliations:** ^1^Department of Sports Medicine, China Medical University, No. 91, Hsueh-Shih Road, Taichung 404402, Taiwan; ^2^Department of Physical Therapy, China Medical University, No. 91, Hsueh-Shih Road, Taichung 404402, Taiwan; ^3^Division of Physical and Health Education, Center for General Education, National Sun Yat-sen University, No. 70 Lienhai Road, Kaohsiung 80424, Taiwan; ^4^Department of Occupational Therapy, Asia University, No. 500, Lioufeng Road, Wufeng District, Taichung 41354, Taiwan; ^5^Department of Physical Therapy and Rehabilitation, Rehabilitation Assistive Device Center, Da-Chien General Hospital, No. 6, Shin Guang Street, Miaoli 36049, Taiwan

## Abstract

*Purpose*. To conduct a meta-analysis and systematic review examining whether Tai Chi Chuan could have mental and physical benefits for patients with knee osteoarthritis.* Methods*. MEDLINE, PUBMED, EMBASE, and CINAHL databases were searched for relevant studies. Data of the studies were collected, and outcomes were classified using the International Classification of Functioning, Disability, and Health model. Effect sizes of the mental and physical components were determined, along with the recommendation grades of Philadelphia Panel Classification System for Tai Chi Chuan on knee osteoarthritis.* Results*. Eleven studies were selected and retrieved from the databases. The results of meta-analysis revealed that the effects of Tai Chi Chuan were observed for physical components in the body functions and structures domain. The effects favoring Tai Chi Chuan were observed in the physical component in the activities and participation domain. Insufficient data was included in the meta-analysis of the mental component.* Conclusions*. The review revealed that Tai Chi Chuan had beneficial outcomes for patients with knee osteoarthritis. The evidence-based results represented that it had small-to-moderate effects on body functions and structures, activities, and participation of physical component. However, there was insufficient evidence to support that Tai Chi Chuan had beneficial mental effect.

## 1. Introduction

The worldwide elderly population was increasing considerably while the prevalence of knee osteoarthritis is increasing among the older adults [1]. Knee osteoarthritis symptoms include joint pain, inflammation, and swelling [2]. Joint discomfort can impede the ability of the older adults to engage in daily and functional activities [1]. Conservative treatments for knee osteoarthritis often involve physical therapy, weight control, and therapeutic exercise [2]. Therapeutic exercise, a widely accepted treatment approach, increases the muscle strength of the thighs, thereby enhancing joint stability and reducing cartilage loss in the knee [3]. Selecting an appropriate form of therapeutic exercise is vital for treating and preventing knee osteoarthritis because high-impact or heavy-load exercises may exacerbate knee osteoarthritis.

Approximately 18% and 27% of male and female older adults suffered from knee osteoarthritis, respectively [4]. Previous study demonstrated that depression had positive correlation with the physical impairment and disability, and the patients with knee osteoarthritis were inclined to feel depressed compared to healthy people [5]. They had high depressed mood and anxiety, because of worrying over worse physical functions and discomfortable symptoms of knee osteoarthritis. The older adults worried at these symptoms and searched for more complementary therapies. Tai Chi Chuan, which is a traditional Chinese martial art, is a common type of palliative low-impact and aerobic exercise [6]. Because it emphasizes body relaxation, rhythmic breathing, and slow motion, Tai Chi Chuan can be appropriate for the older adults intending to perform therapeutic exercise [7]. The results of previous studies had shown that Tai Chi Chuan could improve the balance ability, muscle strength, and cardiopulmonary function for osteoarthritis patients [7, 8] and relieve psychological problems such as depression, anxiety, and tension [9]. Thus, selecting therapeutic exercise appropriate for treating knee osteoarthritis is of interest to the older adults.

Being a palliative therapeutic exercise technique, Tai Chi Chuan includes mind-body exercises and is considered appropriate for older adults [6]. To the best of our knowledge, previous studies of systematic review focused on the evidence to support the effects of Tai Chi Chuan on pain relief and physical function improvement in the patients with knee osteoarthritis [10, 11]. However, empirical medical evidence supporting the efficacy of Tai Chi Chuan in treating knee osteoarthritis was scant, and its mental and physical effects have not been confirmed. In the present study, a systematic review was conducted using a comprehensive meta-analysis for exploring the mental and physical effects of Tai Chi Chuan when managing knee osteoarthritis.

## 2. Methods

### 2.1. Search Strategy

Published articles were searched and related to Tai Chi Chuan and osteoarthritis in the MEDLINE, PUBMED, EMBASE, and CINAHL databases by using “Tai Chi,” “Tai Chi Chuan,” “Tai Ji,” “knee osteoarthritis,” and “osteoarthritis” as the keywords. Inclusion criteria were that the experimental design was a randomized control trial; the experimental and control groups comprised people practicing and not practicing Tai Chi Chuan, respectively; the participants were clinically diagnosed with knee osteoarthritis (i.e., clinical and radiographic evidence of osteoarthritis); and the articles were published in international journals. Two investigators, each with more than 10 years of professional experience in sports medicine, screened and identified the articles which satisfied the criteria. The Jadad Quality Score was used to assess the quality of the articles. The scale comprised five items: whether the experiment was randomized, whether the randomization method was appropriate, whether the participants received blind tests, whether the evaluators conducted blind tests, and whether participant dropouts were recorded [12]. The highest score was 5, indicating the highest quality study. The recruited articles were recorded by the published year, first authors, participant number, movement type, duration and frequency of Tai Chi Chuan practice, and the assessments and outcomes after the intervention. By referring to the International Classification of Functioning, Disability, and Health (ICF), which developed by the World Health Organization in 2001, the assessments for the outcomes of Tai Chi Chuan in treating knee osteoarthritis were classified into two domains: body functions and structures as well as activities and participation [13]. Mental and physical components of the assessments were also categorized, respectively [13].

### 2.2. Data Collection and Meta-Analysis

Meta-analysis was conducted to evaluate the effects of Tai Chi Chuan. Various effects of Tai Chi Chuan were assessed to determine the outcomes. The extracted data were outcomes in terms of means and standard deviations in the experimental and control groups, which were then used to estimate the standardized mean differences (SMDs) and 95% confidence intervals (CIs) to determine the effect size of Tai Chi Chuan. A subgroup analysis for the individual effect size of the outcome variables was performed on the extracted data by using the MedCalc software (MedCalc, Mariakerke, Belgium). Publication bias was determined using Rosenthal's file drawer method, and the fail-safe number was observed to be higher than the tolerance level. *Q* statistic test was used to analyze homogeneity or heterogeneity of the extracted data. The effects of various assessment outcomes were categorized into subgroups by using ICF. The effect sizes were characterized using the method developed by Cohen, and values of 0.2–0.5, 0.5–0.8, and >0.8 were considered small, moderate, and large effect sizes, respectively. Finally, the Philadelphia Panel Classification System was used to analyze the recommendation grades of the evaluated efficacies [14].

## 3. Results

### 3.1. Quantity and Quality of the Articles

**Figure 1:**
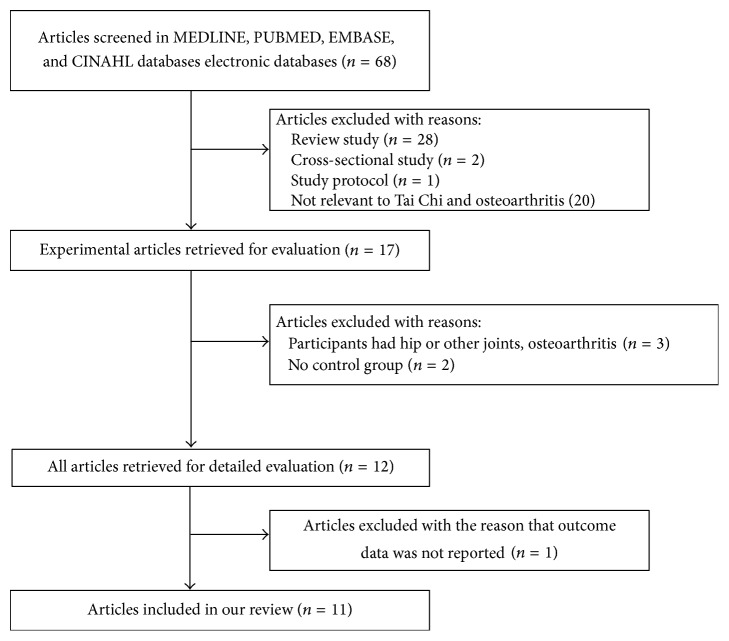
Flow diagram of article search.

After searching the databases, 68 articles were collected; however, 28 review, 2 cross-sectional study, and 1 study protocol articles were excluded (Figure 1). After excluding 20 articles not related to Tai Chi Chuan and osteoarthritis, 17 experimental articles regarding the effects of Tai Chi Chuan on knee osteoarthritis were retrieved. Among these, 6 articles were excluded because participants had osteoarthritis of the hip or other joints, a control group was not included, and outcome data was not reported. Finally, 11 articles were analyzed [15–25]. The recruited articles were published from 2003 to 2015 year, and had high quality scores of 3–5 (Table 1).

### 3.2. Intervention Programs

In the experimental groups, 12–31 movements of Sun-style form and 10–24 movements of Yang-style form were adopted; however, Lee et al. [18] used an unnamed 18-movement form of Tai Chi Chuan (Table 1). Furthermore, 40–65-min training sessions of Tai Chi Chuan class were conducted over 1–4 times each week for 6–24 weeks in all experimental groups. The control groups did not have Tai Chi Chuan programs but received education classes or telephone interviews for the same duration along with the experimental groups. The contents of the education classes and telephone interviews were related to healthy diets, exercise benefits, and the managements of knee osteoarthritis. By contrast, in 2 studies conducted by Song et al. [15] and Lee et al. [18], the control group did not receive any form of intervention.

### 3.3. Effects on Mental and Physical Components

According to the ICF model [13], the assessment methods of the selected articles were classified into the following four categories.

#### 3.3.1. Mental Component in the Body Functions and Structures Domain

A 28-item motivation scale for health behaviors was used to assess perceived self-efficacy, benefits, barriers, and emotional salience [17]. The 5-point self-efficacy scores were also used for assessments [20]. The 60-point Center for Epidemiologic Studies Depression Index and 30-point mini-mental state examination were used to evaluate cognitive and emotional impairment [20, 23].

#### 3.3.2. Physical Component in the Body Functions and Structures Domain

The 10-point visual analog scale and 35-point knee pain scale of Western Ontario and McMaster Universities Osteoarthritis Index (WOMAC) were used to assess the severity of pain during knee movement [15–21, 23, 24]. A verbal descriptive pain scale specific for older adults with cognitive impairment to assess pain-related behaviors during functional activities was also used [25]. The active range of motion for knee movement was assessed through goniometry [16], and the flexibility of the affected knee joint was assessed using the knee stiffness scale of WOMAC [15–21, 24]. An isokinetic dynamometer (Cybex 770, Lumex, USA) was used to measure the knee flexor and extensor strength and endurance at the speeds of 60°/s and 180°/s [15, 22]. Bone mineral density was measured using dual-energy X-ray absorptiometry (GE Lunar PIXImus, Lunar Co., West Indian Federation) [22]. The factors affecting knee joint loading, such as body weight and body mass index (BMI), were also recorded [15, 20, 21, 24].

#### 3.3.3. Mental Component in the Activities and Participation Domain

The 25-item scale for health behaviors comprised the variables of health responsibility, exercise, diet behavior, stress management, and smoking habits [17]. Mental component of the 36-item Short Form Health Survey (SF-36) was used to assess the mental health [18, 20].

#### 3.3.4. Physical Component in the Activities and Participation Domain

Cardiovascular functioning test [15], 6-minute walk test [18, 20, 21, 24], stair climb test [21, 24], sit-to-stand test [20, 23], and timed-up-and-go test [23, 24] were performed to assess physical performance. As the symptoms subsided, the motion frequency and time consumption decreased. The physical function scale of WOMAC [15, 16, 18–21, 23, 24], physical health component of SF-36 [18, 20], and Physical Activity Scale for the Elderly [24] were used to assess physical activity. Furthermore, the Survey of Activities and Fear of Falling in the Elderly was also used to assess the frequency of falling [19, 22].

### 3.4. Review Results of Studies

Analyses of all the included articles revealed that the WOMAC scale scores significantly decreased after Tai Chi Chuan intervention (Table 2), suggesting that the symptoms and physical function of knee osteoarthritis were improved. Wang et al. [20] and Brismée et al. [16] reported that Tai Chi Chuan intervention significantly improved pain assessment results of the experimental group compared with those of the control group. Regarding functional activities and participation, stair climb test [21, 24], timed-up-and-go test [23, 24], and sit-to-stand test [20, 23] scores improved significantly after Tai Chi Chuan intervention in the experimental group compared with those in the control group. Song et al. [19, 22] also reported that the scores of the Survey of Activities and Fear of Falling in the Elderly improved significantly after Tai Chi Chuan intervention. Some studies showed that BMI and body weight decreased significantly in the experimental group [15, 20, 24]. In addition, Wang et al. [20] and Lee et al. [18] revealed that Tai Chi Chuan yielded significant improvements in the physical and mental health components of the SF-36 in the experimental group compared with that in the control group.

### 3.5. Results of Meta-Analysis

**Figure 2:**
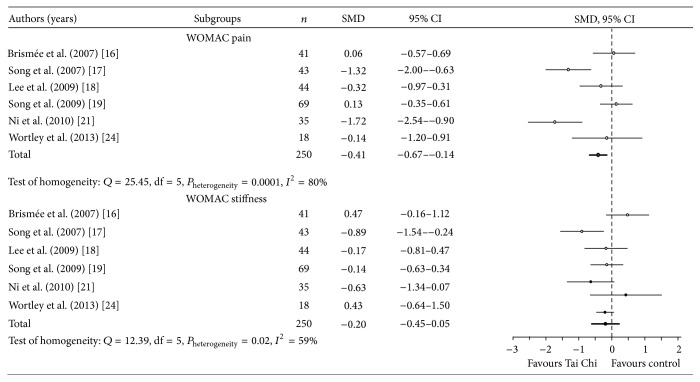
Effect of Tai Chi Chuan on physical component in body functions and structures domain.

**Figure 3:**
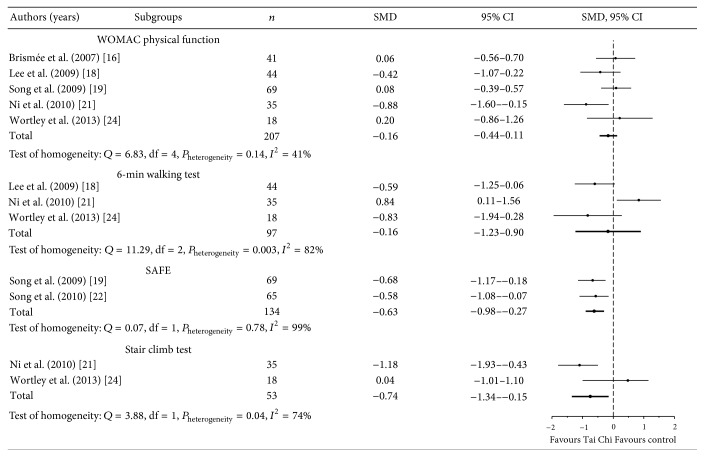
Effect of Tai Chi Chuan on physical component in the activities and participation domain.

In the meta-analysis, the publication bias did not influence the outcomes of data analysis (fail-safe number = 189; tolerance level = 65). Insufficient data was included in the meta-analysis of the mental component in the body functions and structures as well as activities and participation domains. The results of meta-analyses revealed that the effects of Tai Chi Chuan were observed for physical components in the body functions and structures domain: WOMAC pain (total SMD = −0.41; 95% CI = −0.67 to −0.74; and *P*_heterogeneity_ < 0.05) and stiffness (total SMD = −0.20; 95% CI = −0.45 to −0.05; and *P*_heterogeneity_ < 0.05, Figure 2). Small effects were observed in the WOMAC pain and stiffness scale scores among patients with knee osteoarthritis practicing Tai Chi Chuan. The effects favouring Tai Chi Chuan were observed in the physical component in the activities and participation domain: WOMAC physical function (total SMD = −0.16; 95% CI = −0.44 to −0.11; and *P*_heterogeneity_ = 0.14), 6-min walking test (total SMD = −0.16; 95% CI = −1.23 to 0.90; and *P*_heterogeneity_ < 0.05), stair climb test (total SMD = −0.76; 95% CI = −1.34 to 0.15; and *P*_heterogeneity_ < 0.05), and the Survey of Activities and Fear of Falling in the Elderly (total SMD = −0.63; 95% CI = −0.98 to −0.27; and *P*_heterogeneity_ = 0.78, Figure 3); in other words, the meta-analysis results indicated that, after the intervention of Tai Chi Chuan, the patients with knee osteoarthritis showed small-to-moderate effects in WOMAC physical function, 6-min walking test, stair climb test, and Survey of Activities and Fear of Falling in the Elderly. Weak evidence result indicated the effects of Tai Chi Chuan for treating knee osteoarthritis on the change in the mental component, because there was insufficient data to perform a meta-analysis.

## 4. Discussion

### 4.1. Summary of Review Results

The current study revealed that after 8-week to 6-month class training sessions of Tai Chi Chuan, the patients with knee osteoarthritis exhibited improvements in their physiological and psychological states in 11 articles. Fransen et al. [26] indicated that short duration (i.e., <10 weeks) class training of Tai Chi Chuan alleviates knee osteoarthritis pain. By using 8-week class training of Tai Chi Chuan, Lee et al. [18] demonstrated a nonsignificant improvement in the WOMAC scores. Nevertheless, after the class training, significant improvements were observed on the SF-36 and 6-min walking test scores, indicating that Tai Chi Chuan caused potential improvements in muscle endurance. Similar to endurance and aerobic exercises, Tai Chi Chuan comprised rhythmic movements and emphasis on body balance and coordination [19]. Tai Chi Chuan movements involve several postures, such as slight knee bending, arms below the shoulder level, forward or backward strides, and turning around while shifting the center of gravity [17]. Tai Chi Chuan was divided into at least five styles of Tai Chi Chuan, which were Chen-style, Wu-style, W'u-style, Sun-style, and Yang-style forms, according to Chinese martial art styles. Considering the geography and physical conditions of elderly participants, scholars had designed Tai Chi Chuan sets comprising 10–31 movements, the basic components of which resemble moves used in the original styles. The motivation and health behaviors of the elderly can improve by practicing Tai Chi Chuan styles comprising numerous movements [17]. In addition, the muscle strength of lower extremity as well as balance and coordination abilities of patients with knee osteoarthritis could be improved, and the mental capacity of the older adults can be enhanced [18, 20]. By contrast, Tai Chi Chuan set comprising relatively few movements are easy for the elderly to learn, thereby reducing their frustration of failure in learning. The study results of Brismée et al. [16] also indicated that elderly participants practicing 10 movements of Tai Chi Chuan exhibited a high level of acceptance and their dropout rates were low. In addition, Tai Chi Chuan was set as a few movement patterns, which consumed relatively less time during each cycle of the training, specifically for older adults [16]. Therefore, the number of cycles and duration of Tai Chi Chuan must be increased to achieve the positive effects, which is similar to effects of endurance and aerobic exercises. The effect of this factor should be considered when using Tai Chi Chuan as a therapeutic exercise.

Tai Chi Chuan could improve the physical functions of patients with osteoarthritis in terms of performance in the 6-minute walking test, stair climb test, and balance test [18, 20, 21]. Wortley et al. [24] revealed that Tai Chi Chuan effectively improves aerobic capacity, based on 11% and 12% increases in the stair climb test and timed-up-and-go test, respectively. The results indicated that Tai Chi Chuan can enhance the lower extremity muscle strength and cardiopulmonary function. Ni et al. [21] indicated that Tai Chi Chuan can increase the quadriceps muscle strength of the patients with osteoarthritis; thus alleviating quadriceps weakness was the main factor causing knee osteoarthritis. Wang et al. [20] and Lee et al. [18] also reported that Tai Chi Chuan was a low-intensity and low-impact exercise and could improve the motor functions of patients with osteoarthritis. Song et al. [15, 22] investigated the effects of Tai Chi Chuan on the strength and endurance of knee extensor and flexor muscles. They reported that Tai Chi Chuan enhanced the knee extensor endurance of patients with knee osteoarthritis; however, the difference in the strength of the knee extensor and flexor muscles between the experimental and control groups was nonsignificant. Tai Chi Chuan involved full-weight-bearing movement, in which an individual applies loading and uploading patterns in various directions [22]. In addition, while maintaining core stability, shifting the center of gravity requires the use of knee extensor endurance for maintaining postural balance [20, 23]. Therefore, Tai Chi Chuan could improve knee extensor endurance to manage knee osteoarthritis.

Obesity could increase the severity of knee osteoarthritis, and Tai Chi Chuan was beneficial for reducing the body weight of patients with knee osteoarthritis. Ni et al. [21] indicated that Tai Chi Chuan training produces effects similar to those of aerobic sports. This is specifically because the patients practicing Tai Chi Chuan engage in a wide range of motions which involve large muscle groups, along with calorie consumption as the metabolic rates of the body are increased, thereby resulting in weight loss. These factors were vital for relieving osteoarthritis-induced pain. Nevertheless, Wang et al. [20] reported that no weight loss was observed after patients with knee osteoarthritis practiced Tai Chi Chuan. Nevertheless, the osteoarthritis-induced knee pain was relieved. It indicated that weight loss was not the mechanism through which Tai Chi Chuan training reduces pain. A comparison revealed that the research design of Wang et al. [20] comprised a 12-week, 10-movement Tai Chi Chuan training, whereas that of Ni et al. [21] consisted of a 24-week, 24-movement training of Tai Chi Chuan. The difference in training intensity and duration may have contributed to the difference in the observed results and ultimately different conclusions. Among the 11 studies examined, only Song et al. [22] investigated the effects of Tai Chi Chuan training on bone density. Tai Chi Chuan practice for 6–12 months could slow bone density loss [27, 28]. By contrast, Song et al. [22] observed that Tai Chi Chuan training lasting only 6 months also had similar effects on slowing bone density loss. Tai Chi Chuan could reduce bone density loss because it involves weight-bearing movement [29]. According to Wolf's law, Tai Chi Chuan involved changes in postures resulting in forces similar to the foot-floor impact force generated while walking [30]. When bones are subjected to pressure, bone cell proliferation occurs, thereby reducing bone loss. Because Tai Chi Chuan can improve the metabolism, patients must continue Tai Chi Chuan for a longer duration. Specifically, patients with knee osteoarthritis must practice Tai Chi Chuan for at least 6 months to achieve considerable reduction in bone loss.

### 4.2. Evidence on Physical and Mental Component Effects of Tai Chi Chuan

The most crucial benefit of Tai Chi Chuan training was the increase in quadriceps muscle strength, and enhanced lower extremity strength can aid in preventing or reducing daily function loss and deterioration in the elderly [24]. Song et al. [22] also indicated that Tai Chi Chuan can effectively decrease fear of falling during daily functional activities of patients with knee osteoarthritis and that the positive effects of Tai Chi Chuan were attributed to increases in the muscle strength and endurance of the lower extremity. Improved daily life functions enable the elderly to readily interact with society, thereby enhancing self-efficacy and social function [17, 20]. This indicated a high efficacy of Tai Chi Chuan training in alleviating the psychological symptoms and depression resulting from knee osteoarthritis. Furthermore, patients with knee osteoarthritis were prone to a high risk of falling because of knee deformation and insufficient lower extremity muscle strength [19, 22]. By enhancing the endurance of the quadriceps, Tai Chi Chuan training could improve the posture control ability in the older adults, thereby reducing the risk of falling [22]. Numerous studies had confirmed that engaging in endurance sports can reduce the risks of falling in the older adults, increase self-care abilities, decrease mental illnesses, and increase social interaction frequency. Tai Chi Chuan training had similar effects to mind-body exercises [17, 20, 24, 25]. Furthermore, Tai Chi Chuan is also a low-impact endurance exercise and thus is appropriate for patients with knee osteoarthritis. The results of meta-analyses revealed that Tai Chi Chuan had small effect on the body functions and structures and small-to-moderate effects on the activities and participation domain of the physical component. Hence, on the basis of the improvements in the physical component of patients with knee osteoarthritis, a Philadelphia Panel Classification System level B was assigned to Tai Chi Chuan because of the significant improvements, with clinical significance, observed after the intervention.

Knee osteoarthritis was an inflammation resulting from knee degeneration, and knee cartilage damage may result in knee inflammation and pain [31]. The results of our meta-analysis indicated that Tai Chi Chuan could alleviate osteoarthritis-induced pain. However, the mechanism of the analgesic effectiveness of Tai Chi Chuan training remained unknown. On the basis of their evidence-based results, Bennell and Hinman [32] attributed the analgesic effectiveness to Tai Chi Chuan being a low-impact exercise, which increased lower extremity muscle strength. Tai Chi Chuan training could improve cardiopulmonary capacity, balance ability, and functional performances through increased muscle strength of lower extremity [15, 18, 20, 21, 24]. A significant correlation had been reported between weak lower extremity muscle strength and the pain experienced by patients with knee osteoarthritis [33]. Various subsequent assessments for evaluating the mental effects of Tai Chi Chuan had shown beneficial outcomes. Due to the lack of consistent assessments and result data for meta-analysis, insufficient evidence indicated that Tai Chi Chuan had mental component effects on improvements in body functions and structures or activities and participation domains for knee osteoarthritis.

Tai Chi Chuan was considered a form of mind and body component that enables people to maintain the mind and body balance [17, 20]. Subsequently, the functions of the immune and autonomic nervous systems can be improved and regulated, thereby achieving pain relief effects. Wang et al. [20] reported that Tai Chi Chuan is a form of rhythmic exercise, in which people achieve enhanced self-efficacy by regulating breathing and sustaining mind and body balance. These findings can aid in disrupting the “pain cycle” experienced by patients with knee osteoarthritis. Currently, the mechanism underlying the effects of Tai Chi Chuan training on knee osteoarthritis had not been identified. However, reduced pain enables patients to continue exercising, thereby increasing muscle strength of lower extremity and improving abilities of balance and coordination and functions of daily life. At the psychological level, reduced pain was beneficial for achieving emotional stability and a balanced mood [25]. Hence, although the exact mechanism underlying its knee osteoarthritis pain reduction remains unknown, Tai Chi Chuan had significant positive effects for alleviating knee osteoarthritis symptoms. But there was insufficient data performing a meta-analysis to support the mental effects. On the basis of the mental component of participants with knee osteoarthritis, Tai Chi Chuan was assigned a Philadelphia Panel Classification System level C+ because of the significant improvements, without clinical significance, observed after the intervention.

### 4.3. Limitations and Suggestions for Future Research

This study had some limitations in our review. In the subgroup analysis, insufficient assessment items could be classified, and, hence, the meta-analysis could be performed using only a few studies. Although the various outcomes were presented and analyzed in the subgroup, the effects may be influenced by the data of few articles. Furthermore, a main limitation of the subgroup analysis based on ICF model was that effects on mental and components were not analyzed for lack of data in the recruited articles. There are also several suggestions for further investigations. First, the study results demonstrated that Tai Chi Chuan training has significant physiological and psychological effects on patients with knee osteoarthritis because Tai Chi Chuan could reduce osteoarthritis-induced pain and increase the quadriceps endurance. However, the interaction of biomechanical mechanism of Tai Chi Chuan and psychological outcomes of patients with knee osteoarthritis has not been identified. Second, no studies have explored exercise intensity, duration, and frequency appropriate for patients with knee osteoarthritis. Because of the long duration and complex movements involved in Tai Chi Chuan, participant dropouts are common; therefore, further studies on the need for exercise among older adults are necessary. Finally, several of the 11 analyzed studies included small sample sizes and limited long term follow-up outcomes. Most studies are concerned with assessments of Tai Chi Chuan in the physical component, but very few are concerned with the assessments of the mental component.

## 5. Conclusions

In summary, Tai Chi Chuan had beneficial outcomes for patients with knee osteoarthritis, that is, improving knee extensor endurance, aerobic capacity, and body balance and coordination and reducing the body weight and bone density loss. Positive effects can be observed in the physical component in body functions and structures as well as activities and participation domains. There was insufficient evidence to support that Tai Chi Chuan had beneficial mental effect on patients with knee osteoarthritis, because of insufficient data in the recruited articles. Consequently, future studies could emphasize mental effects of Tai Chi in patients with knee osteoarthritis. More studies with large samples and a long term follow-up were also suggested to be conducted.

## Figures and Tables

**Table 1:** Summary of intervention programs in the included articles.

Author (year)	*n*	Age	Experimental groups	Tai Chi sessions	Control groups	Sessions	Jadad
Song et al. (2003) [15]	43	63.65	12 movements of Sun-style form (*n* = 22)	60 min class program, 3 times each week for 12 weeks	No intervention (*n* = 21)	No session	3
Brismée et al. (2007) [16]	41	69.80	24 movements of Yang-style form (*n* = 22)	40 min class program, 3 times each week for 6 weeks 40 min home-based program, 3 times each week for 6 weeks	Education class (*n* = 19)	40 min class program, 3 times each week for 6 weeks	5
Song et al. (2007) [17]	43	63.65	12 movements of Sun-style form (*n* = 22)	60 min class program, 3-4 times each week for 12 weeks	Interview (*n* = 21)	15–20 min, 2 times	4
Lee et al. (2009) [18]	44	68.55	18 movements (*n* = 29)	60 min class program, 2 times each week for 8 weeks	No intervention (*n* = 15)	No session	4
Song et al. (2009) [19]	69	61.15	31 movements of Sun-style form (*n* = 30)	60 min class program, 2 times each week for the first 3 weeks and 1 time each week for the next 6 months	Education class (*n* = 39)	120 min class program, 1 time each month for 6 months	3
Wang et al. (2009) [20]	40	65.50	10 movements of Yang-style form (*n* = 20)	60 min class program, 2 times each week for 12 weeks	Education class (*n* = 20)	60 min class program, 2 times each week for 12 weeks	4
Ni et al. (2010) [21]	35	63.18	24 movements of Yang-style form (*n* = 18)	45 min class program, gradually increased from 2 to 4 times each week for 24 weeks	Education class (*n* = 17)	45 min class program, 1 time each week for 24 weeks	5
Song et al. (2010) [22]	65	62.11	31 movements of Sun-style form (*n* = 30)	55–65 min class program, 1 time each week for 6 months 20 min home-based program, every day for 6 months	Education class (*n* = 35)	120 min class program, 1 time each month for 6 months	3
Tsai et al. (2013) [23]	55	78.91	12 movements of Sun-style form (*n* = 28)	40 min class program, 3 times each week for 20 weeks	Education class (*n* = 27)	40 min class program, 3 times each week for 20 weeks	4
Wortley et al. (2013) [24]	18	69.30	12 movements of Yang-style form (*n* = 12)	60 min class program, 2 times each week for 10 weeks	Telephone interview (*n* = 6)	1 time	3
Tsai et al. (2015) [25]	55	79.01	12 movements of Sun-style form (*n* = 28)	20–40 min class program, 3 times each week for 20 weeks	Education class (*n* = 27)	20–40 min class program, 3 times each week for 20 weeks	5

**Table 2:** Summary of assessments and outcomes in the included articles.

Author (year)	Assessments	Outcomes	Adverse effects
Song et al. (2003) [15]	WOMAC (pain/stiffness/physical function); knee extensor strength and endurance; flexibility; BMI; cardiovascular functioning test	Knee extensor strength and endurance, flexibility, cardiovascular functioning test were improved^*∗*^ BMI and WOMAC were decreased^*∗*^	No adverse event
Brismée et al. (2007) [16]	WOMAC (pain/stiffness/physical function); VAS; knee range of motion	WOMAC and VAS were decreased^*∗*^	No adverse event
Song et al. (2007) [17]	WOMAC (pain/physical function); motivation; health behaviors	Motivation and health behaviors were improved^*∗*^ WOMAC was decreased^*∗*^	No adverse event
Lee et al. (2009) [18]	WOMAC (pain/stiffness/physical function); SF-36; 6-min walking test	SF-36 and 6-min walking test were improved^*∗*^ WOMAC was decreased	No adverse event
Song et al. (2009) [19]	WOMAC (pain/stiffness/physical function); Survey of Activities and Fear of Falling in the Elderly	WOMAC was decreased Survey of Activities and Fear of Falling in the Elderly was improved^*∗*^	No adverse event
Wang et al. (2009) [20]	WOMAC (pain/stiffness/physical function); VAS; SF-36; BMI; 6-min walking test; sit-to-stand test; Center for Epidemiology Studies Depression Index; self-efficacy	6-min walking test, sit-to-stand test, SF-36, self-efficacy were improved^*∗*^ WOMAC, VAS, Center for Epidemiology Studies Depression Index were decreased^*∗*^	One participant reported an increase of knee pain
Ni et al. (2010) [21]	WOMAC (pain/stiffness/physical function); 6-min walking test; stair climb test; body weight	6-min walking test, stair climb test, body weight were improved^*∗*^ WOMAC was decreased^*∗*^	No adverse event
Song et al. (2010) [22]	Bone mineral density; knee extensor and flexor strength and endurance; Survey of Activities and Fear of Falling in the Elderly	Knee extensor endurance was increased^*∗*^ Bone mineral density and Survey of Activities and Fear of Falling in the Elderly were improved^*∗*^	No adverse event
Tsai et al. (2013) [23]	WOMAC (pain/stiffness/physical function); timed-up-and-go test; sit-to-stand test; mini-mental state examination	Timed-up-and-go test, sit-to-stand test, mini-mental state examination were improved^*∗*^ WOMAC was decreased^*∗*^	No adverse event
Wortley et al. (2013) [24]	WOMAC (pain/stiffness/physical function); BMI; 6-min walking test; timed-up-and-go test; stair climb test; Physical Activity Scale for the Elderly	Physical Activity Scale for the Elderly, 6-min walking test, stair climb test, timed-up-and-go test^*∗*^ were improved WOMAC was decreased	No adverse event
Tsai et al. (2015) [25]	Verbal descriptive scale; pain behavior	Verbal descriptive scale and pain behavior were decreased^*∗*^	No adverse event

^*∗*^ *P* < 0.05, significant differences between before and after Tai Chi Chuan. WOMAC, Western Ontario and McMaster Universities Osteoarthritis Index; BMI, body mass index; VAS, Visual Analog Scale; and SF-36, 36-item Short Form Health Survey.
